# Multiple Sampling and Interpolation in Weighted Fock Spaces of Entire Functions

**DOI:** 10.1007/s11785-020-01065-4

**Published:** 2021-02-16

**Authors:** Luis Alberto Escudero, Antti Haimi, José Luis Romero

**Affiliations:** 1 Acoustics Research Institute, Austrian Academy of Sciences, Wohllebengasse 12-14, 1040 Vienna, Austria; 2 Faculty of Mathematics, University of Vienna, Oskar-Morgenstern-Platz 1, 1090 Vienna, Austria

**Keywords:** Sampling, Fock space, Interpolation, Derivative, Bargmann–Fock space

## Abstract

We characterize sampling and interpolating sets with derivatives in weighted Fock spaces on the complex plane in terms of their weighted Beurling densities.

## Introduction

### Results and Context

Let $\phi :\mathbb {C}\rightarrow \mathbb {R}$ be a subharmonic function with Laplacian bounded above and below by positive constants. The *weighted Fock space* of entire functions is

$$\begin{aligned} \mathcal {F}_{\phi }^{2} := \left\{ f:\mathbb {C}\rightarrow \mathbb {C}: f \text{ holomorphic, } \Vert f\Vert _\phi ^2 :=\int _{{\mathbb {C}}}|f(z)|^{2} e^{-\phi (z)} dA(z)<\infty \right\} , \end{aligned}$$

where $dA$ denotes the Lebesgue measure on $\mathbb {C}$. We study sampling and interpolation on weighted Fock spaces, where we sample or interpolate using not only the function values, but also the values of its derivatives. Such process is sometimes called multiple or Hermite sampling and interpolation. While sampling and interpolation theory provides a mathematical foundation for the tasks of digitalization and encoding of analog signals into bit-streams, the use of derivatives incorporates the *trend* of the signal as well, and is well studied in Paley–Wiener spaces and shift-invariant spaces on the real line; see, e.g., [1, 2, 12, 21].

In this article, we characterize the sets that allow for multiple sampling and interpolation in weighted Fock spaces in terms of certain densities, provided that the number of derivatives considered at each sampling point is bounded. The precise assumptions and definitions are given in Sect. 2.

The prime example of a Fock space is the *Bargmann–Fock* space, where $\phi (z)=\alpha |z|^2$, and $\alpha > 0$ [26]. For this weight, the Heisenberg group acts irreducibly on $\mathcal {F}_{\phi }^{2}$, which results in a important homogeneity property called *translation invariance* [26, Section 2.6]. More precisely, $\mathcal {F}_{\phi }^{2}$ is invariant under the so-called *Bargmann–Fock* shifts [26]:

1.1 $$\begin{aligned} f(z) \mapsto e^{-\tfrac{\alpha }{2} |w|^2 + \alpha z {\bar{w}}} f(z-w), \qquad w \in {\mathbb {C}}. \end{aligned}$$

Necessary and sufficient conditions for sampling and interpolation without derivatives are fully described in terms of planar Beurling densities [23, 24]. Specifically, the lower and upper Beurling densities of a set $\Lambda \subseteq {\mathbb {C}}$ are

1.2 $$\begin{aligned} D^-(\Lambda )&:= \liminf _{R \longrightarrow \infty } \inf _{z \in {\mathbb {C}}} \frac{\# \left( \Lambda \cap B(z,R) \right) }{\pi R^2}, \end{aligned}$$

1.3 $$\begin{aligned} D^+(\Lambda )&:= \limsup _{R \longrightarrow \infty } \sup _{z \in {\mathbb {C}}} \frac{\# \left( \Lambda \cap B(z,R) \right) }{\pi R^2}, \end{aligned}$$

where *B*(*z*, *R*) denotes the Euclidean ball with center *z* and radius *R*. For $\phi (z)=\alpha |z|^2$ and a *separated* set $\Lambda$, the existence of two sampling constants $A,B>0$ leading to a sampling inequality

1.4 $$\begin{aligned} A\Vert f\Vert _{\phi }^{2} \le \sum _{\lambda \in \Lambda } \left| {f(\lambda )}\right| ^{2} e^{-\phi (\lambda )} \le B\Vert f\Vert _{\phi }^{2}, \qquad f \in \mathcal {F}_{\phi }^{2}, \end{aligned}$$

is completely characterized by the density condition $D^-(\Lambda ) > \frac{\alpha }{\pi }$. Similarly, the density condition $D^+(\Lambda ) < \frac{\alpha }{\pi }$ completely characterizes the validity of the following interpolation property: given a sequence $\{c_\lambda : \lambda \in \Lambda \} \subset {\mathbb {C}}$ satisfying the growth condition $\sum _{\lambda \in \Lambda } |c_\lambda |^2 e^{- \phi (\lambda )} < \infty$ there exists (at least) one function $f \in \mathcal {F}_{\phi }^{2}$ such that

1.5 $$\begin{aligned} f(\lambda )=c_\lambda , \qquad \lambda \in \Lambda . \end{aligned}$$

Slightly more technical formulations of these density characterizations apply to arbitrary, possibly non-separated sets [23, 24]. These results parallel Beurling’s sampling and interpolation theorems for the Paley–Wiener space of square integrable functions on the real line having Fourier transforms supported on the unit interval, with the difference that the latter results do not provide a complete characterization in terms of densities, as the necessary density condition involves a non-strict inequality while the sufficient involves a strict one [6, 7].^1^ Translation invariance plays a key role in [23, 24], and the arguments do not seem to extend easily to more general weights.

For the classical weight $\phi (z)=\alpha |z|^2$, sampling and interpolation with bounded multiplicities are studied in [9], while unbounded multiplicities are treated in [8]. In this setting the sampling (1.4) and interpolation equations (1.5) involve not only the function *f* but also its *translation covariant derivative*

1.6 $$\begin{aligned} f(z) \mapsto \alpha {\bar{z}}f(z) - \partial f(z) \end{aligned}$$

applied iteratively. The operator (1.6) is called covariant, because it commutes with the Bargmann–Fock shifts (1.1). When considering multiplicities, the characterization of sampling and interpolation is in terms of weighted variants of the planar Beurling densities (1.2), (1.3), where each point $\lambda$ is counted multiply, according to the number of derivatives that are evaluated at $\lambda$ [9] (see Sect. 2.5 for more details.).

Sampling and interpolation on Fock spaces with general weights has been studied in [5] and [19]. The characterization is in terms of variants of the Beurling densities (1.2), (1.3), where the factor $\pi R^2$ is replaced by the weighted measure

1.7 $$\begin{aligned} \int _{B(z,R)} \Delta \phi \,dA = \int _{B(z,R)} \partial {\bar{\partial }} \phi \,dA. \end{aligned}$$

As $\partial {\bar{\partial }} \big [ \alpha |z|^2 \big ] = \alpha$, Beurling densities defined using (1.7) extend the classical ones (1.2), (1.3) up to normalization. For general weights, the lack of translation invariance demands new techniques, such as the introduction of *approximate translation operators* [19], and $\bar{\partial }$-surgery together with Riesz decompositions [5] (see Sect. 3).

In this article we simultaneously extend the results from [5, 9, 19] to study sampling and interpolation with multiplicities on Fock spaces with general weights. Our starting point is the observation that, for the classical weight $\phi (z)=\alpha |z|^2$, the covariant derivative (1.6) is the formal adjoint of the $\bar{\partial }$-operator with respect to the scalar product that induces the norm of $\mathcal {F}_{\phi }^{2}$. Explicitly,1.8We consider this operator for general weights $\phi$, and study sampling and interpolation involving iterated applications of $\bar{\partial }^*_\phi$. We derive a characterization of these properties in terms of a suitable variant of the Beurling densities (1.2), (1.3), that counts points multiply according to the number of concerned derivatives as in [9], and weights balls with the Laplacian factor (1.7) as in [5, 19].

### Motivation

One main motivation for our results is validating the differential operator (1.8) as an adequate replacement for the covariant derivative (1.6), by showing that it leads to similar sampling and interpolation properties. Other candidates for this role are the differential operators defined as ordinary differentiation at the origin, conjugated with the approximate translation operators from [19]. This second choice is however unnatural, as it arbitrarily distinguishes the origin, and leads to complicated compatibility issues (stemming from the fact that approximate translation operators are not associated with a group representation). On the other hand, although they lack the simplicity of (1.8), differential operators associated with approximate translations would allow a more straightforward generalization of the proofs of [5, 19]. In any case, one can use our present results to show a posteriori that both differential operators - (1.8) and the ones defined in terms of approximate translations - do lead to similar sampling and interpolation theorems.

Fock spaces find important applications in the simultaneous time-frequency analysis of real variable functions. The most successful applications concern the classical weights and *Gabor systems*, which are structured functional dictionaries for $L^2({\mathbb {R}})$,

1.9 $$\begin{aligned} {\mathcal {G}}(g,\Lambda ) =\big \{ e^{2 \pi i b \cdot } g(\cdot - a) : \lambda =(a,b) \in \Lambda \big \} \end{aligned}$$

generated by the Gaussian function $g(t) = e^{-\pi t^2}$, $t \in {\mathbb {R}}$, and their *time-frequency shifts* along a given set of nodes $\Lambda \subseteq {\mathbb {R}}^2$. By means of the *Bargmann transform*, the spanning properties of (1.9) can be reformulated as sampling and interpolation properties of $\Lambda$ on the Fock space with weight $\phi (z)=\pi |z|^2$ [10], and thus characterized in terms of Beurling densities [18, 23, 24]. Multiple sampling and interpolation on $\mathcal {F}_{\phi }^{2}$ concerns in turn the spanning properties of *multi-window Gabor systems*, where the Gaussian function is supplemented with additional Hermite functions [9, Remark 1]. Other weights $\phi (z)=\alpha \pi |z|^2$ allow a similar analysis for the general Gaussian function $g(t) = e^{-a t^2}$. Applications of more general weighted Fock spaces to time-frequency analysis are more recent, and mainly connected to non-linear properties of the so-called short-time Fourier transform, where for example weights are chosen in a signal dependent fashion, see e.g., [13]. We expect our current results to find applications in this direction.

### Organization and Technical Overview

In Sect. 2 we present the precise definitions and assumptions as well as the main results. Sect. 3 provides useful estimates from potential theory, which are important to treat derivatives of functions in Fock spaces. The sufficiency of the density conditions for sampling and interpolation is shown under additional technical assumptions in Sect. 4, following closely the arguments from [5]. The most important task here is showing that the operator (1.8) is suitably compatible with that line of argument, which is based on Hörmander’s $L^2$-estimates for ${\overline{\partial }}$ [16]. The necessity of the density conditions for multiple sampling and interpolation in the weighted case is challenging, as the classical approach relies on translation invariance [9], and the one in [19] on a subtle form of approximate translation invariance. Instead of adapting the delicate proof from [19], in Sect. 5 we develop a perturbation argument that allows us to reduce the problem to the case of no multiplicities. This line of reasoning seems to provide a more direct proof of the necessary density conditions for multiple sampling and interpolation even for the classical weights [9]. The main results are finally proved in Sect. 6, where the remaining technical assumptions are removed. Section A is an appendix listing auxiliary results related to iterated derivatives of composite functions.

## Definitions and Main Results

### Sets with Multiplicity

A *set with multiplicities* is a pair $\left( \Lambda , m_{\Lambda }\right)$, where $\Lambda \subseteq \mathbb {C}$, and $m_{\Lambda } : \Lambda \rightarrow {\mathbb {N}}$ is a bounded function called *multiplicity function*. In problems concerning evaluations of functions at $\Lambda$, the number $m_{\Lambda }(\lambda )$ indicates how many derivatives are involved at the point $z=\lambda$. More precisely, $m_\Lambda (\lambda )=k$ indicates the interpolation or sampling of a function and its first $k-1$ derivatives at $\lambda$.

### Notation

Complex disks are denoted $B(z,r):=\{y\in \mathbb {C}: |z-y|< r \}$, with $z\in \mathbb {C}$ and $r>0$. We use the notation $x\lesssim y$ if there exists a constant such that $x\le C y$. The constant is normally allowed to depend on $\sup _{\lambda \in \Lambda } m_{\Lambda }(\lambda )$ and $\phi$, while other dependencies are noted explicitly. The precise value of unspecified constants may vary from line to line.

For a set with multiplicities $(\Lambda , m_{\Lambda })$, we denote by $\ell _\phi ^2(\Lambda , m_\Lambda )$ the space of sequences $a \equiv \big (a_{(\lambda ,j)} \big )_{\lambda \in \Lambda ,\, 0\le j \le m_{\Lambda }(\lambda )-1 }$ of complex numbers with finite norm:

$$\begin{aligned}\Vert a\Vert _{\ell _\phi ^2(\Lambda ,m_\Lambda )}^2 = \sum _{\lambda \in \Lambda } \sum _{j=0}^{m_{\Lambda }(\lambda )-1} |a_{(\lambda ,j)}|^2 e^{-\phi (\lambda )}. \end{aligned}$$

When $m_\Lambda \equiv 1$ we simply write $\ell _\phi ^2(\Lambda )$.

### Separated Sets

A set $\Lambda \subseteq \mathbb {C}$ is called *relatively separated* if

$$\begin{aligned}{\text {rel}}(\Lambda ):=\sup \left\{ \#\left( \Lambda \cap B(z,1)\right) : z \in {\mathbb {C}}\right\} <\infty , \end{aligned}$$

and it is called *(uniformly) separated* if

$$\begin{aligned}\rho (\Lambda ):=\inf \left\{ \left| \lambda -\lambda ^{\prime }\right| : \lambda \ne \lambda ^{\prime } \in \Lambda \right\} >0. \end{aligned}$$

Separated sets are relatively separated, and relatively separated sets are finite unions of separated sets. For simplicity of notation, we write $\rho$ instead of $\rho (\Lambda )$ when no confusion can arise. We say that a set with multiplicities $(\Lambda , m_\Lambda )$ is separated (respectively relatively separated) if $\Lambda$ is separated (respectively relatively separated).

### Wirtinger Derivatives and the  Operator

We normalize the Laplacian as

$$\begin{aligned}\Delta :=\frac{1}{4}\left( \partial _{x}^{2}+\partial _{y}^{2}\right) =\partial \bar{\partial }, \end{aligned}$$

where $\partial$ and $\bar{\partial }$ denote the Wirtinger derivatives:

$$\begin{aligned} \partial =\frac{1}{2}\left( \partial _{x}-i\partial _{y}\right) \quad \text{ and } \quad \bar{\partial }=\frac{1}{2}\left( \partial _{x}+i\partial _{y}\right) . \end{aligned}$$

For $f\in C^{1}(\mathbb {C})$ we define the operatorwhich is the formal adjoint of the $\bar{\partial }$-operator with respect to the weighted scalar product

$$\begin{aligned}\langle f, g\rangle _{\phi }=\int f(z) \overline{g(z)} e^{-\phi (z)} dA(z). \end{aligned}$$

When the dependence on $\phi$ is clear from the context, we write for short . For the classical weight $\phi (z)=\alpha |z|^2$,  is the covariant derivative (1.6).

### Interpolating Sets and Sampling Sets

We say that $\left( \Lambda , m_{\Lambda }\right)$ is an* interpolating set *for $\mathcal {F}_{\phi }^{2}$, if for any sequence $c\in \ell _\phi ^2{(\Lambda ,\,m_{\Lambda })}$ there exists a function $f \in \mathcal {F}_{\phi }^{2}$ such that , for all $\lambda \in \Lambda$ and $j\in \{0,\ldots ,{m_{\Lambda }(\lambda )-1}\}$. We say that $\left( \Lambda , m_{\Lambda }\right)$ is a *sampling set* for $\mathcal {F}_{\phi }^{2}$, if there exist constants $A, B>0$ such thatFor the classical weight $\phi (z)=\alpha |z|^2$, using the covariance property of ${\overline{\partial }}^*_{\phi }$, it is easy to see thatwhere $e_j(z)=\left( \alpha ^{j+1} / {\pi }\right) z^j$ and $W_w$ denotes the Bargmann–Fock shift by *w* (1.1). Multiple sampling and interpolating sets with respect to the weight $\phi (z)=\alpha |z|^2$ are defined in [9] in terms of Bargmann–Fock shifts of the normalized monomials $f_j(z) = (\alpha ^j / j!)^{1/2} z^j$. Since, as in [9], we only consider sets with bounded multiplicity, for the classical weight $\phi (z)=\alpha |z|^2$, the present notion of multiple sampling and interpolation is equivalent to the one from [9].

### Beurling Densities

Given a set with multiplicities $\left( \Lambda , m_{\Lambda }\right)$,  is defined as the number of points of the set $\Lambda$ in the disk *B*(*z*, *r*), counted with multiplicities. That is,where

$$\begin{aligned} \nu = \nu _\Lambda :=\sum _{\lambda \in \Lambda } m_\Lambda (\lambda ) \cdot \delta _{\lambda } \end{aligned}$$

is a weighted sum of Dirac deltas, and $1_{B(0, r)}$ is the characteristic function of the disk. The lower and upper Beurling densities of $\left( \Lambda , m_{\Lambda }\right)$ with respect to $\phi$ are:2.12.2

### Compatibility Assumptions

We always assume that sets with multiplicities have bounded multiplicity functions and denote

$$\begin{aligned} {n_\Lambda } := \sup _{\lambda \in \Lambda } m_{\Lambda }(\lambda ) -1. \end{aligned}$$

We say that $(\Lambda , m_\Lambda )$ and $\phi$ are *compatible* if the following conditions hold. For ${n_\Lambda }=0$ or ${n_\Lambda }=1$, we only require that $m<\Delta \phi <M$ for some positive constants *m*, *M*. For ${n_\Lambda }\ge 2$, we additionally require $\phi \in C^{{n_\Lambda }+1}({\mathbb {C}})$ and $\sup _{z\in \mathbb {C}} |\partial _z^{j} \Delta \phi (z) |< \infty$, for all $j=1, \ldots ,{n_\Lambda }-1$.

The conditions are chosen so that there exists $\varepsilon _r>0$ for which

2.3 $$\begin{aligned} \left| \partial _z^j\left( \int _{B(\zeta ,r)}\log |w-z|\ \Delta \phi (w)\, dA(w)\right) \right| \le C_r, \end{aligned}$$

for every $\zeta \in \mathbb {C}$, $z\in B(\zeta , \varepsilon _r)$ and $0\le j \le {n_\Lambda }$.

### Main Results

Our goal is to derive the following characterization of sampling and interpolating sets.

#### Theorem A

Let $(\Lambda , m_\Lambda )$ be a set with multiplicities that is compatible with the weight $\phi$. Then $(\Lambda , m_\Lambda )$ is interpolating for $\mathcal {F}_{\phi }^{2}$ if and only if it is separated and satisfies $D_{\phi }^{+}(\Lambda , m_\Lambda )<1/\pi {}$.

#### Theorem B

Let $(\Lambda , m_\Lambda )$ be a set with multiplicities that is compatible with the weight $\phi$. Then $(\Lambda , m_\Lambda )$ is sampling for $\mathcal {F}_{\phi }^{2}$ if and only if it is relatively separated and $\Lambda$ contains a separated subset $\Lambda ^\prime$ satisfying .

## Riesz Decomposition

The Riesz decomposition, presented below, describes the structure of subharmonic functions on bounded subdomains of the complex plane. It is an essential tool to study weighted Fock spaces, as their very definition involves a subharmonic function.

### Theorem 3.1

(Riesz decomposition) Let *D* be a domain in ${\mathbb {C}}$, and let $u:D\rightarrow {\mathbb {R}}$ be a subharmonic function. If $K\subseteq D$ is an open and relatively compact set, then there exists a harmonic function *h* on *K* such that

$$\begin{aligned}u(z)=h (z)+G[\Delta u](z), \qquad z \in K, \end{aligned}$$

where

3.1 $$\begin{aligned} G[\Delta u](z) =\frac{2}{\pi } \int _{K}\log |w-z|\ \Delta u(w)\, dA(w).\end{aligned}$$

The function $G[\Delta u]$ in (3.1) is called *the logarithmic potential* of $\Delta u$ on the domain *K*. For more on Riesz decomposition, see [14, 20, 22].

We recall the following fact from potential theory, (see, e.g., [25, Thm. 10]).

### Lemma 3.2

Let $\Omega \subset {\mathbb {R}}^{n}$ be open and bounded. Let $B_{1}=B\left( x_{0}, R\right)$, $B_{2}=B\left( x_{0}, 2R\right) \subset \bar{B_2} \subset \Omega$ be concentric balls. Suppose that *u* solves $\Delta u=f$ in $\overline{B_{2}}$, and $f \in C^{0}(\Omega )$, then $u \in C^{1, \alpha }(B_1)$ for any $\alpha \in (0,1),$ and

$$\begin{aligned} \Vert u\Vert _{C^{1, \alpha }\left( B_1\right) } \lesssim \Vert f\Vert _{L^\infty (B_2)}+\Vert u\Vert _{L^{2}(B_2)}. \end{aligned}$$

Here,

$$\begin{aligned} \Vert u\Vert _{C^{k,\alpha }(\Omega )}:=\sum _{0\le \left| \beta \right| \le k} \Vert \partial ^\beta u\Vert _{L^\infty (\Omega )} + \sup _{\left| \beta \right| =k} \sup _{\begin{array}{c} x,y\in \Omega \\ x\ne y \end{array}} \frac{\left| D^\beta u (x)-D^\beta u (y) \right| }{\left| x-y \right| ^\alpha }. \end{aligned}$$

As an application, we have the following bounds for Riesz decomposition.

### Lemma 3.3

Let $(\Lambda , m_\Lambda )$ and $\phi$ be compatible, and let $\varepsilon > 0$. For each $\lambda \in \Lambda$, let

$$\begin{aligned}\phi (z)=h_\lambda (z)+G[\Delta \phi ](z), \qquad z \in B(\lambda ,\varepsilon ), \end{aligned}$$

be the Riesz decomposition of $\phi$ on $B(\lambda ,\varepsilon )$, where $h_\lambda$ is harmonic and $G[\Delta \phi ]$ is the logarithmic potential of $\Delta \phi$ on $B(\lambda ,\varepsilon )$. Write $h_\lambda = 2 {\text {Re}} H_\lambda$, where $H_\lambda$ is holomorphic, and set $G_{\lambda }=H_\lambda -H_\lambda (\lambda )$.

Then there exists $C_\varepsilon > 0$, such that for $z\in B(\lambda ,\varepsilon )$,

3.2 $$\begin{aligned} \left| G[\Delta \phi ](z) \right|&\le C_\varepsilon , \end{aligned}$$

3.3 $$\begin{aligned} \left| \phi (z)-\phi (\lambda )-2{\text {Re}} G_{\lambda }(z)\right|&\le C_\varepsilon , \end{aligned}$$

and if $z\in B(\lambda ,\varepsilon /{2^{n_\Lambda }})$ and $1\le k\le {n_\Lambda }$,

3.4 $$\begin{aligned} |\partial ^{k} G[\Delta \phi ](z)|&\le C_\varepsilon , \end{aligned}$$

3.5 $$\begin{aligned} \left| \partial ^k(G_{\lambda } - \phi )(z) \right|&\le C_\varepsilon . \end{aligned}$$

The constant $C_\varepsilon$ depends on $\varepsilon$ but not on $\lambda$.

### Proof

Let $z\in B(\lambda , \varepsilon )$. We first note that

$$\begin{aligned}\big |G[\Delta u](z)\big |&\le \frac{2}{\pi } \int _{B(\lambda ,\varepsilon )}\big |\log |w-z|\big |\ \big |\Delta u(w)\big |\, dA(w) \\&\le \frac{2}{\pi } \sup _{w\in \mathbb {C}} |\Delta \phi (w)| \int _{B(0,2\varepsilon )}\big |\log |w|\big |\ \, dA(w) =: C_\varepsilon ^{(1)}. \end{aligned}$$

Note also that

3.6 $$\begin{aligned} \phi (z)-\phi (\lambda )-2{\text {Re}} G_{\lambda }(z) = G[\Delta \phi ](z)-G[\Delta \phi ](\lambda ). \end{aligned}$$

Consequently,

3.7 $$\begin{aligned} \left| \phi (z)-\phi (\lambda )-2{\text {Re}} G_{\lambda }(z)\right| =\left| G[\Delta \phi ](z)-G[\Delta \phi ](\lambda )\right| < 2 C_\varepsilon ^{(1)}. \end{aligned}$$

We now show similar bounds for the derivatives of the logarithmic potential of $\Delta \phi$. Suppose $n_\Lambda \ge 1$. As shown in [11, Lemma 4.1], since $\Delta \phi$ is bounded and integrable in $B(\lambda ,\varepsilon /2)$, $G[\Delta \phi ]\in C^1(\lambda ,\varepsilon /2)$. Moreover, as consequence of [11, Eq. 4.8] for any $z\in B(\lambda ,\varepsilon /2)$,

3.8 $$\begin{aligned} \left| \partial G[\Delta \phi ](z) \right| \le \frac{2}{\pi } \sup _{w\in \mathbb {C}}|{\Delta \phi }(w)| \int _{B(0,2\varepsilon )} \left| {w} \right| ^{-1} dA(w) =C_\varepsilon ^{(2)}.\end{aligned}$$

Suppose now that $n_\Lambda \ge 2$. Recall that in this case, $\phi$ is assumed to be $C^{n_{\Lambda }+1}$, thus the Laplacian is $C^{n_{\Lambda }-1}$. By Theorem 3.1,

3.9 $$\begin{aligned} \Delta \left( G[\Delta \phi ]\right) =\Delta \phi ,\end{aligned}$$

on $B(\lambda ,\varepsilon )$. Taking derivatives in (3.9) we obtain $\Delta \partial G[\Delta \phi ]=\partial \Delta \phi$. On account of Lemma 3.2, since $\partial \Delta \phi \in C(B(\lambda ,\varepsilon ))$, we obtain that $\partial G[\Delta \phi ] \in C^1(B(\lambda ,\varepsilon /4))$ and that there exists a constant $\tilde{C_2}>0$ for which

$$\begin{aligned}\Vert \partial ^2 G[\Delta \phi ]\Vert _{L^\infty (B(\lambda ,\varepsilon /4))} \le \tilde{C_2} \left( \Vert \partial \Delta \phi \Vert _{L^\infty ( B(\lambda ,\varepsilon /2) )}+\Vert \partial G[\Delta \phi ]\Vert _{L^{2}(B(\lambda ,\varepsilon /2))}\right) , \end{aligned}$$

where the right-hand of the last inequality is uniformly bounded as a consequence of (3.8) and the assumption that $\partial \Delta \phi$ is uniformly bounded. Iterating this argument, we obtain that

$$\begin{aligned}&\Vert \partial ^k G[\Delta \phi ]\Vert _{L^\infty (B(\lambda ,\varepsilon /2^k))} \\&\quad \le \tilde{C_k} \left( \Vert \partial ^{k-1} \Delta \phi \Vert _{L^\infty ( B(\lambda ,\varepsilon /2^{k-1}) )}+\Vert \partial ^{k-1} G[\Delta \phi ]\Vert _{L^{2}(B(\lambda ,\varepsilon /2^{k-1}))}\right) , \end{aligned}$$

where the right-hand is uniformly bounded. This proves (3.4).

Finally, since *G* is holomorphic, repeated differentiation of (3.6) yields,

3.10 $$\begin{aligned} \partial ^k G_{\lambda } (z) - \partial ^k\phi (z) = \partial ^k [ 2 {\text {Re}} G_{\lambda }] (z) - \partial ^k\phi (z) = - \partial ^k G[\Delta \phi ] (z). \end{aligned}$$

Thus (3.4), gives (3.5). $$\square $$

The following lemma helps profit from Riesz decomposition in estimates involving derivatives.

### Lemma 3.4

Let $(\Lambda , m_\Lambda )$ and $\phi$ be compatible, $D\subseteq \mathbb {C}$ a domain, $K \subseteq D$ open and relatively compact, and $f,H:D\longrightarrow \mathbb {C}$ analytic functions. Let $G[\Delta \phi ]$ be as in Theorem 3.1. Then there exist a constant $C=C_{\phi , K}$, such that for every $\lambda \in \Lambda \cap K$, and $0 \le j \le n_\Lambda$, the following estimates hold:

(i) $$\displaystyle |\partial ^j(fe^{-H})(\lambda ) |^2 \le C \sum _{k=0}^j |\partial ^k ( fe^{-H-G[\Delta \phi ]}) (\lambda ) |^2 ,$$
(ii) $$\displaystyle |\partial ^j(fe^{-H-G[\Delta \phi ]})(\lambda ) |^2 \le C \sum _{k=0}^j |\partial ^k ( fe^{-H}) (\lambda ) |^2 .$$

### Proof

By Leibniz rule:

$$\begin{aligned} \partial ^j(fe^{-H})=\partial ^j(fe^{-H-G[\Delta \phi ]+G[\Delta \phi ]})&= \sum _{k=0}^{j} \left( {\begin{array}{c}j\\ k\end{array}}\right) \partial ^{k} (f e^{-H-G[\Delta \phi ]}) \partial ^{j-k}(e^{G[\Delta \phi ]}). \end{aligned}$$

By Lemma 3.3, $\partial ^{j-k}(e^{G[\Delta \phi ]})$ is bounded for $0\le k\le j$. This yields (i); (ii) follows similarly. $\square$

Finally, we note that Cauchy bounds extend to weighted derivatives.

### Lemma 3.5

Let $\left( \Lambda , m_{\Lambda }\right)$ be a separated set with multiplicities that is compatible with $\phi$. Then for each $\lambda \in \Lambda$ and $j \le {n_\Lambda }$,

### Proof

For each $\lambda \in \Lambda$ we write $\phi (z)=h_\lambda (z)+G[\Delta \phi ](z)$ as in Theorem 3.1 where the decomposition is taken on the set $B(\lambda ,1)$. We apply Lemma 3.4, together with Cauchy’s bound to obtain$\square$

## Sufficient Conditions for Interpolation and Sampling

Throughout this section we assume that $(\Lambda , m_\Lambda )$ is a separated set with multiplicities that is compatible with $\phi$.

### Interpolation

We start by showing that interpolation can be solved locally.

#### Lemma 4.1

Let $\varepsilon >0$, $\lambda \in \Lambda$ and $\{c_l:\, l=0, \ldots , m_\Lambda (\lambda )-1 \} \subseteq \mathbb {C}$. Then there exists $f_\lambda : B(\lambda ,\varepsilon ) \longrightarrow \mathbb {C}$ analytic such thatfor all $z\in B(\lambda ,\varepsilon )$.

#### Proof

We consider $G_\lambda$ as in the Lemma 3.3 on the set $B(\lambda ,\varepsilon )$. We define

4.1 $$\begin{aligned} f_{\lambda }(z) := p_\lambda (z) e^{G_{\lambda }(z)}, \end{aligned}$$

where

4.2 $$\begin{aligned} p_\lambda (z) := \sum _{j=0}^{m_{\Lambda }(\lambda )-1} \frac{k_j}{j!} (z-\lambda )^j, \end{aligned}$$

and $k_j$ are real coefficients to be chosen so that4.3for all $j\in \{0,\dots ,m_\Lambda (\lambda )-1\}$.

To obtain an explicit formula of $k_j$ we proceed as follows,4.4The *n*-th derivative of two composite functions can be computed by means of *Faà di Bruno’s* formula and the *Bell polynomials* (see Eq. (A.6) and Section A for definitions and suitable references). More precisely,

4.5 $$\begin{aligned} \partial ^{k} e^{G_{\lambda }-\phi }(w)=e^{G_{\lambda }(w)-\phi (w)} B_k\bigg ( \partial (G_{\lambda }-\phi )(w),\ \ldots \ ,\ \partial ^k (G_{\lambda }-\phi )(w) \bigg ), \end{aligned}$$

where $B_k$ is the $k-$th complete Bell polynomial. We write $B_{k}^{g}(w):=B_{k}\left( \partial g(w), \ldots , \partial ^{k} g(w)\right)$ to shorten notation.

Since $G_\lambda (\lambda )=0$, substituting (4.5) into (4.4), and evaluating at $w=\lambda$, it follows thatTherefore, (4.3) is equivalent to:

4.6 $$\begin{aligned} k_j = (-1)^j c_{j} - {\sum _{l=0}^{j-1} \left( {\begin{array}{c}j\\ l\end{array}}\right) \ k_{l} }\ B_{j-l}^{G_{\lambda }-\phi }(\lambda ), \end{aligned}$$

for all $j\in \{0,\dots ,m_\Lambda (\lambda )-1\}$. Solving the recursion yields:

4.7 $$\begin{aligned} k_j = {\sum _{l=0}^{j} (-1)^l \left( {\begin{array}{c}j\\ l\end{array}}\right) \ c_{l} }\ B_{j-l}^{\phi - G_{\lambda }}(\lambda ), \end{aligned}$$

for all $j\in \{0,\dots ,m_\Lambda (\lambda )-1\}$; see Appendix A.3 for a proof.

#### Remark 4.1

By Lemma 3.3, $\left| \partial ^j(\phi -G_{\lambda })(\lambda ) \right| \le C_\varepsilon$ for each $j\in \{1,\ldots ,m_\Lambda (\lambda )-1\}$. Since $B_{j}^{\phi -G_{\lambda }}(\lambda )$ is a polynomial evaluated at $\Big (\partial (\phi -G_{\lambda })(\lambda ),\, \ldots ,\, \partial ^j(\phi -G_{\lambda })(\lambda )\Big )$, we have $\left| B_{j}^{\phi -G_{\lambda }}(\lambda )\right| \lesssim C_\varepsilon$.

On account of the above remark we conclude that

$$\begin{aligned}|k_j|^2 \lesssim C_\varepsilon {\sum _{l=0}^{j} \ |c_{l}|^2 }. \end{aligned}$$

By Lemma 3.3, we finally obtain

$$\begin{aligned} \left| f_{\lambda }(z)\right| ^{2} e^{-\phi (z)}=\left| p_{\lambda }(z)\right| ^{2} e^{2{\text {Re}}{G_\lambda (z)}-\phi (z)}&\lesssim C_\varepsilon \left( {\sum _{l=0}^{m_{\Lambda }(\lambda )-1} \left| c_{l}\right| ^{2}}\right) e^{-\phi (\lambda )}. \end{aligned}$$

$$\square $$

For technical reasons we now study the interpolation problem on $\mathcal {F}_{\phi }^{2}$ with respect to the operator ${\bar{\partial }}_{{\tilde{\phi }}}^{*}$ associated with a second weight ${\tilde{\phi }}$. Provided that both weights are sufficiently smooth, powers of both operators are formally related by4.8

#### Proposition 4.2

Let $\left( \Lambda , m_{\Lambda }\right)$ be a separated set with multiplicities that is compatible with $\phi$. Assume additionally that $\Delta \phi$ is continuous. Let $\chi _{r}=\frac{1}{\pi r^{2}} 1_{B(0, r)}$ and $\nu :=\sum _{\lambda \in \Lambda } m_\Lambda (\lambda ) \cdot \delta _{\lambda }$. Suppose that there exists $\delta >0$ and $r>0$ such that

4.9 $$\begin{aligned} \pi \nu \star \chi _{r}(z)< \Delta \phi (z)-\delta , \end{aligned}$$

for all $z\in {\mathbb {C}}$. Let ${\tilde{\phi }}: {\mathbb {C}} \rightarrow {\mathbb {R}}$ satisfy

4.10 $$\begin{aligned}&\Vert \partial ^{j} (\phi - {\tilde{\phi }})\Vert _\infty < \infty , \qquad 0 \le j \le n_\Lambda \end{aligned}$$

4.11 $$\begin{aligned}&0< \inf _{z \in {\mathbb {C}}} \Delta {\tilde{\phi }}(z) \le \sup _{z \in {\mathbb {C}}} \Delta {\tilde{\phi }}(z) < \infty . \end{aligned}$$

Then $\left( \Lambda , m_{\Lambda }\right)$ solves the following interpolation problem with respect to ${\tilde{\phi }}$: given $c\in \ell _\phi ^2{(\Lambda ,\,m_{\Lambda })}$ there exists a function $f \in \mathcal {F}_{\phi }^{2}$ such that $\,{\bar{\partial }}_{{\tilde{\phi }}}^{*}\, ^{(j)} f(\lambda )=c_{(\lambda ,j)}$, for all $\lambda \in \Lambda$ and $j\in \{0,\dots ,{m_{\Lambda }(\lambda )-1}\}$.

(Note that, by (4.10) and (4.11), $\ell _\phi ^2{(\Lambda ,\,m_{\Lambda })}=\ell _{{\tilde{\phi }}}^2{(\Lambda ,\,m_{\Lambda })}$ and $\mathcal {F}_{\phi }^{2}=\mathcal {F}_{{\tilde{\phi }}}^{2}$, while ${\tilde{\phi }}: {\mathbb {C}} \rightarrow {\mathbb {R}}$ is also compatible with $\left( \Lambda , m_{\Lambda }\right)$. )

#### Proof

Let $c \in \ell _\phi ^2(\Lambda ,\,m_{\Lambda })$. As in [5] we construct a weight that has singularities on $\Lambda$. Let $E(z)=\frac{1}{\pi } \log |z|^{2}$ and

$$\begin{aligned} v=E \star \left( \nu -\nu \star \chi _{r}\right) . \end{aligned}$$

Let $\psi :=\phi +\pi v .$ The modified weight $\psi$ has the properties

$$\begin{aligned} \Delta \psi&\ge \pi \nu +\delta \ge \delta , \\ \psi&\le \phi , \end{aligned}$$

and for each $\lambda \in \Lambda$ the following inequality holds

4.12 $$\begin{aligned} |\psi (z)- m_\Lambda (\lambda ) \log | z-\lambda |^{2}-\phi (z) | \le C_{\rho }, \end{aligned}$$

when $z\in B(\lambda , \rho / 2)$, for some constant $C_\rho$ that depends on $\rho (\Lambda )$ and *r*, although this second dependency is not stressed in the notation. (Notice the factor $m_\Lambda (\lambda )$ in front of the logarithm.)

The first step is to construct the non-analytic interpolant. Fix $\lambda \in \Lambda$. Note first that, by (4.10) and (4.11), $(\Lambda , m_\Lambda )$ is compatible with ${\tilde{\phi }}$. By Lemma  4.1 applied to the weight ${\tilde{\phi }}$, there is an analytic function $f_\lambda :B(\lambda ,\rho /2) \longrightarrow \mathbb {C}$ such that for each $j\in \{0,\ldots ,m_\Lambda (\lambda )-1\}$,

$$\begin{aligned} \,{\bar{\partial }}_{{\tilde{\phi }}}^{*}\, ^{(j)} f_\lambda (\lambda )&= c_{(\lambda ,j)}, \end{aligned}$$

and, due to (4.10), for all $z \in B(\lambda ,\rho /2)$

4.13 $$\begin{aligned} \left| f_\lambda (z)\right| ^2 e^{-\phi (z)}&\le C_\rho \sum _{j=0}^{m_{\Lambda } (\lambda )-1} |c_{(\lambda ,j)}|^2 e^{-\phi (\lambda )}, \end{aligned}$$

where the constant $C_\rho$ is independent of $\lambda$.

Now we patch these functions together. We let $g \in C_{0}^{\infty }$ be 1 on $B(0, \rho / 4)$ and 0 outside $B(0, \rho / 2)$, satisfying $|{\overline{\partial }} g|<C_{\rho }^{'}$. Then

$$\begin{aligned} f(z)=\sum _{\lambda \in \Lambda } f_{\lambda }(z) g(z-\lambda ) \end{aligned}$$

solves the interpolation problem, although this function might not be analytic.

We consider the problem ${\overline{\partial }} u= {\overline{\partial }}f$. By Hörmander’s estimate, there exists a solution satisfying

$$\begin{aligned}&\int _{{\mathbb {C}}}|u(z)|^{2} e^{-\phi (z)} dA(z) \\&\quad \le \int _{{\mathbb {C}}}|u(z)|^{2} e^{-\psi (z)} dA(z) \lesssim \int _{{\mathbb {C}}}|{\overline{\partial }} f(z)|^{2} e^{-\psi (z)} dA(z) \\&\quad \lesssim \int _{{\mathbb {C}}}|{\overline{\partial }} f(z)|^{2} e^{-\phi (z)} dA(z) \lesssim \sum _{\lambda \in \Lambda }{\sum _{l=0}^{m_{\Lambda }(\lambda )-1} \left| c_{(\lambda ,l)}\right| ^{2}} e^{-\phi (\lambda )}<\infty . \end{aligned}$$

For the first inequality we used that $\psi \le \phi$. For the second we used that *f* is the solution provided by Hörmander’s estimate^2^ and that $\Delta \psi >\delta .$ For the third, we used (4.12) and the fact that ${\overline{\partial }} f$ is zero on a $\rho / 4$ -neighborhood of $\Lambda$. The last inequality follows from the separation of $\Lambda$ and (4.13).

The function *u* is analytic on $B(\lambda , \rho / 4)$, because ${\overline{\partial }} u= {\overline{\partial }}f = 0$ on $B(\lambda , \rho / 4)$. In addition, we estimate

$$\begin{aligned}&\int _{B(\lambda ,\rho /4)}\left| \frac{u(z)}{(z-\lambda )^{m_\Lambda (\lambda )}}\right| ^{2} dA(z) = \int _{B(\lambda ,\rho /4)}|u(z)|^{2} e^{-m_\Lambda (\lambda ) \log |z-\lambda |^{2}} dA(z) \\&\qquad \le C_\rho \sup _{w\in B(\lambda ,\rho /4)} e^{\phi (w)} \int _{B(\lambda ,\rho /4)}|u(z)|^{2} e^{-\psi (z)} dA(z) \\&\qquad \le C_\rho \sup _{w\in B(\lambda ,\rho /4)} e^{\phi (w)} \int _{{\mathbb {C}}}|u(z)|^{2} e^{-\psi (z)} dA(z) <\infty , \end{aligned}$$

and conclude that $\partial ^{j} u(\lambda ) = 0$, for each $\lambda \in \Lambda$ and $0\le j\le m_\Lambda (\lambda )-1$. Moreover,

$$\begin{aligned} {\bar{\partial }}_{{\tilde{\phi }}}^{*}\, ^{(j)}u(\lambda )= & {} (-1)^j e^{{\tilde{\phi }}(\lambda )}\partial ^j\left( u e^{-{\tilde{\phi }}} \right) (\lambda ) \\= & {} (-1)^j e^{{\tilde{\phi }}(\lambda )}{\left[ \sum _{l=0}^{j} \left( {\begin{array}{c}j\\ l\end{array}}\right) \partial ^{ l} u (\lambda ) \partial ^{ j-l}\left( e^{-{\tilde{\phi }}} \right) (\lambda ) \right] }=0. \end{aligned}$$

Thus, the function $f-u$ is holomorphic, belongs to $\mathcal {F}_{\phi }^{2}$ and solves the desired interpolation problem with respect to ${\bar{\partial }}^*_{{\tilde{\phi }}}$. $\square$

### Sampling

#### Proposition 4.3

Let $\left( \Lambda , m_{\Lambda }\right)$ be a separated set with multiplicities that is compatible with $\phi$. Assume additionally that $\Delta \phi$ is continuous. Let $\chi _{r}=\frac{1}{\pi r^{2}} 1_{B(0, r)}$ and $\nu :=\sum _{\lambda \in \Lambda } m_\Lambda (\lambda ) \cdot \delta _{\lambda }$. Suppose that there exists $\delta >0$ and $r>0$ such that

$$\begin{aligned} \pi \nu \star \chi _{r}(z) > \Delta \phi (z)+\delta , \end{aligned}$$

for all $z\in {\mathbb {C}}$. Then $(\Lambda , m_\Lambda )$ is a sampling set for $\mathcal {F}_{\phi }^{2}$.

#### Proof

Let $0<\varepsilon < \min \left( 1,\rho /2\right)$ and $0<t<1$. We follow again [5]. We construct the weight exactly as they do, except that we add each point mass $m_\Lambda (\lambda )$ times at $\lambda$. We write

$$\begin{aligned} \tilde{\nu }(z) := t \sum _{\lambda \in \Lambda } \frac{m_\Lambda (\lambda )}{\pi \varepsilon ^{2}} 1_{B(0, \varepsilon )}(z-\lambda ). \end{aligned}$$

Since $\Delta \phi$ is bounded, we can choose *t* so close to 1 that

$$\begin{aligned} \pi \tilde{\nu } \star \chi _{r}>\Delta \phi +\delta / 2 . \end{aligned}$$

We let $E(z)=\frac{1}{\pi } \log |z|^{2}$, $v=E \star \left( \tilde{\nu }-\chi _{r} \star \tilde{\nu }\right)$ and $\psi =\phi +\pi v .$ Note that

$$\begin{aligned} E \star \tilde{\nu }(z)=\sum _{\lambda \in \Lambda }\ \frac{t \ m_\Lambda (\lambda )}{\left( \pi \varepsilon \right) ^2} \int _{B(\lambda ,\varepsilon )}\log {|z-w|^2} dA(w), \end{aligned}$$

and for $E \star \tilde{\nu } \star \chi _{r}$ we obtain a similar expression. Using that $\log |z-w|^2$ is harmonic on $w\in B(\lambda ,\varepsilon )$ when $d(z,\lambda )>r+\varepsilon$ and that the set is relatively separated, we obtain that $|v| \le C_\varepsilon$. Moreover,

$$\begin{aligned} \phi -C_{\varepsilon } \le \psi \le \phi \end{aligned}$$

and

$$\begin{aligned} \left| \psi -m_\Lambda (\lambda )\ t \log \varepsilon ^{2}-\phi \right| \le C_r \end{aligned}$$

on $B(\lambda , \varepsilon )$. Notice the factor $m_\Lambda (\lambda )$ in front of the logarithm.

Let $h\in \mathcal {F}_{\phi }^{2}$. As shown in [5, Eq. 3], we have

$$\begin{aligned} \delta /2 \int _{\mathbb {C}}|h(z)|^{2} e^{-\psi (z)} dA(z) \le \int _{\mathbb {C}}|h(z)|^{2} e^{-\psi (z)} d \tilde{\nu }(z) . \end{aligned}$$

Thus,

$$\begin{aligned} \int _{\mathbb {C}}|h(z)|^{2} e^{-\phi (z)} dA(z) \lesssim C_r \sum _{\lambda \in \Lambda } m_\Lambda (\lambda ) \frac{\varepsilon ^{-2 m_\Lambda (\lambda ) t}}{\varepsilon ^{2}} \int _{B(\lambda , \varepsilon )}|h(z)|^{2} e^{-\phi (z)}dA(z). \end{aligned}$$

Let $g_{\lambda }=h e^{-G_{\lambda }},$ where $G_{\lambda }$ is as in Lemma 3.3, with respect to the set $B(\lambda ,1)$. Then

$$\begin{aligned} \int _{B(\lambda , \varepsilon )}|h(z)|^{2} e^{-\phi (z)}dA(z) \lesssim \int _{B(\lambda , \varepsilon )}\left| g_{\lambda }(z)\right| ^{2} e^{-\phi (\lambda )} dA(z). \end{aligned}$$

We now use the $(m_\Lambda (\lambda )-1)$-th order Taylor expansion of $g_\lambda$ on $B(\lambda , \varepsilon )$

4.14 $$\begin{aligned} \left| g_{\lambda }(z)\right| ^{2} \lesssim \sum _{j=0}^{m_\Lambda (\lambda )-1} \varepsilon ^{2j}\left| \partial ^{j} g_{\lambda }(\lambda )\right| ^{2} + \varepsilon ^{2 m_\Lambda (\lambda )} \sup _{z \in B(\lambda , \varepsilon )}\left| \partial ^{m_\Lambda (\lambda )} g_{\lambda }(z)\right| ^{2} . \end{aligned}$$

We estimate the $m_\Lambda (\lambda )$-th term by Cauchy estimate

$$\begin{aligned} \sup _{z \in B(\lambda , \varepsilon )}\left| \partial ^{m_\Lambda (\lambda )} g_{\lambda }(z)\right| ^{2} e^{-\phi (\lambda )} \lesssim \int _{B(\lambda , 1)}|h(z)|^{2} e^{-\phi (z)} dA(z). \end{aligned}$$

The remaining terms in (4.14) are estimated observing that for any $w \in B(\lambda , \varepsilon )$

$$\begin{aligned} \partial ^{j} g_{\lambda }(w) = \partial ^{j} (h e^{-\phi } e^{\phi -G_\lambda }) (w) = e^{\phi (w)-G_\lambda (w)} \sum _{k=0}^{j} \left( {\begin{array}{c}j\\ k\end{array}}\right) \partial ^{k}( h e^{-\phi })(w) \cdot B_{j-k}^{\phi -G_\lambda }(w), \end{aligned}$$

where $B_j$ is the $j-$th Bell polynomial. Evaluating at $\lambda$ we obtain:Thus, for each $j \le m_\Lambda (\lambda )-1$, and applying Remark 4.1 we havePutting everything together, we obtain4.15Since $1 \le m_{\Lambda }(\lambda )$,

$$\begin{aligned}\varepsilon ^{2 m_{\Lambda }(\lambda ) (1-t)} \le \varepsilon ^{2 (1-t)}. \end{aligned}$$

The relative separateness of $\Lambda$ implies

$$\begin{aligned} \sum _{\lambda \in \Lambda } \varepsilon ^{2 m_{\Lambda }(\lambda ) (1-t)} \int _{B(\lambda ,1)}|h|^{2} e^{-\phi } dA&\le \varepsilon ^{2 (1-t)} \sum _{\lambda \in \Lambda } \ \int _{B(\lambda ,1)} |h|^{2} e^{-\phi } dA\\&\le \varepsilon ^{2 (1-t)} {\text {rel}}(\Lambda )\, \int _{\mathbb {C}} |h|^{2} e^{-\phi } dA. \end{aligned}$$

Therefore,Choosing $\varepsilon$ small enough, the last term can be absorbed into the left-hand side, yielding the desired sampling estimate. $\square$

## Necessary Conditions for Interpolation and Sampling

In this section we reduce the problem of deriving necessary conditions for interpolation and sampling with derivatives to the corresponding problem without derivatives. We assume throughout this section that $\left( \Lambda , m_{\Lambda }\right)$ is a separated set with multiplicities that is compatible with the weight $\phi$.

Our arguments are based on inspecting Taylor expansions. The following observation will be used repeatedly.

### Remark 5.1

Let $\varepsilon \in (0,1/4)$, $\lambda \in \mathbb {C}$ and $F:U\subseteq \mathbb {C}\longrightarrow \mathbb {C}$ be holomorphic, where $B(\lambda ,1)\subseteq U$. The Taylor expansion of *F* of degree *n* at $\lambda$,

$$\begin{aligned}F(z)=\sum _{k= 0}^{n} \frac{F^{(k)}(\lambda )}{k!}(z-\lambda )^k+E_n(z), \end{aligned}$$

satisfies

5.1 $$\begin{aligned} |E_{n}(z)| \lesssim \varepsilon ^{n+1}\int _{B(\lambda ,1)} |F|\, dA, \qquad |z-\lambda | \le \varepsilon . \end{aligned}$$

In particular, if $\lambda '$ is such that $|\lambda -\lambda '|=\varepsilon$ and $w \in \mathbb {C}$ is such that

$$\begin{aligned} F^{(n)}(\lambda )=\frac{n!}{(\lambda '-\lambda )^n}\left( w -\sum _{k= 0}^{n-1} \frac{F^{(k)}(\lambda )}{k!}(\lambda '-\lambda )^k \right) , \end{aligned}$$

then

5.2 $$\begin{aligned} |F(\lambda ')-w|^2 \lesssim \varepsilon ^{2(n+1)}\int _{B(\lambda ,1)} |F|^2 dA. \end{aligned}$$

As a first step towards the necessary conditions we compute derivatives of weighted functions.

### Lemma 5.1

Let $f\in \mathcal {F}_{\phi }^{2}$, $\lambda \in \Lambda$, and $\varepsilon >0$. We write $\phi =h_{\lambda }+G[\Delta \phi ]$ on $B(\lambda ,\varepsilon )$ as in Lemma 3.3. $H_\lambda :B(\lambda ,\varepsilon ) \longrightarrow \mathbb {C}$ is a holomorphic function such that $2{\text {Re}}{H_\lambda }=h_\lambda$. Then5.3

### Proof

Since $$\partial (e^{-\overline{H_\lambda }})=\partial (\overline{e^{-H_\lambda }})=\overline{\left( {\overline{\partial }} {e^{-H_\lambda }} \right) }\equiv 0,$$$$\square $$

### Interpolation

We now show that a weighted interpolating set can be modified to reduce its maximal multiplicity while preserving its density and interpolating property.

#### Proposition 5.2

Let $\left( \Lambda , m_{\Lambda }\right)$ be a separated set with multiplicities that is compatible with $\phi$. Suppose that $(\Lambda ,m_{\Lambda })$ is interpolating for $\mathcal {F}_{\phi }^{2}(\mathbb {C})$, and that $\sup _{\lambda \in \Lambda } m_{\Lambda }(\lambda ) = {n_\Lambda }+1 \ge 2$. Then there exists a separated and interpolating set $({\tilde{\Lambda }},m_{{\tilde{\Lambda }}})$ such that $\sup _{\lambda \in \tilde{\Lambda }} m_{\tilde{\Lambda }}={n_\Lambda }$ and $D^{\pm }(\Lambda , m_{\Lambda })=D^{\pm }({\tilde{\Lambda }}, m_{{\tilde{\Lambda }}})$.

#### Proof

**Step 1**. *(Definition of the new set).*

Let $0<\varepsilon <\min \{\rho (\Lambda )/2,1/4\}$. We define

$$\begin{aligned} \Lambda _{\text {max}} := \left\{ \lambda \in \Lambda \, : \, m_{\Lambda }(\lambda )=\sup _{z\in \Lambda } m_{\Lambda }(z)={n_\Lambda }+1 \right\} . \end{aligned}$$

For each $\lambda \in \Lambda _{\text {max}}$ we choose $\lambda '\in \mathbb {C}$ such that $|\lambda -\lambda '|=\varepsilon$. We define $\Lambda '=\{\lambda ' : \lambda \in \Lambda _{\text {max}} \}$. Since $\varepsilon < \rho (\Lambda )/2$, it is clear that the map $\lambda \mapsto \lambda '$ is injective and that $\lambda '\not \in \Lambda$.

Now we consider the set $\tilde{\Lambda }=\Lambda \cup \Lambda '$ and the function $m_{{\tilde{\Lambda }}}:\tilde{\Lambda }\longrightarrow \mathbb {N}$ defined by

$$\begin{aligned} m_{{\tilde{\Lambda }}}(z) = {\left\{ \begin{array}{ll} m_{\Lambda }(z) &{}\quad \text {if }z\in \Lambda \text { and } m_{\Lambda }(z)\le {n_\Lambda },\\ {n_\Lambda } &{}\quad \text {if }z\in \Lambda \text { and } m_{\Lambda }(z)={n_\Lambda }+1,\\ 1 &{}\quad \text {if }z\in \Lambda '.\\ \end{array}\right. } \end{aligned}$$

Since $\varepsilon < \rho (\Lambda )/2$ it follows easily that ${\tilde{\Lambda }}$ is separated. It is also clear that $\sup m_{{\tilde{\Lambda }}}={n_\Lambda }$.

**Step 2**. We show that if ${\tilde{a}} \in \ell _\phi ^2({\tilde{\Lambda }},\,m_{{\tilde{\Lambda }}})$, then there exists $f\in \mathcal {F}_{\phi }^{2}$ satisfying:

**(Q.1)**: $$\Vert f\Vert _{\mathcal {F}_{\phi }^{2}}\le C_\Lambda \varepsilon ^{-{n_\Lambda }}\Vert {\tilde{a}}\Vert _{\ell _\phi ^2({\tilde{\Lambda }},\,m_{{\tilde{\Lambda }}})}$$,
**(Q.2)**
, for each $\lambda \in \Lambda$ and $0\le j \le {\min \{{n_\Lambda }-1, m_\Lambda (\lambda )-1 \}}$,
**(Q.3)**: $$\Vert f - {\tilde{a}}_{(\cdot , 0)}\Vert _{\ell _{\phi }^2(\Lambda ')}\le C_\Lambda \varepsilon ^{{n_\Lambda }+1} \Vert f\Vert _{\mathcal {F}_{\phi }^{2}}.$$^3^

To prove the claim, for each $\lambda \in \Lambda$ we write $\phi =h_{\lambda }+G[\Delta \phi ]$ on $B(\lambda ,1)$ as in Theorem 3.1. Since $h_\lambda$ is harmonic, there exists $H_\lambda :B(\lambda ,1) \longrightarrow \mathbb {C}$ analytic such that $2{\text {Re}}{H_\lambda }=h_\lambda$.

For each $\lambda \in \Lambda _{\text {max}}$ we let $b_\lambda$ be defined by

5.4 $$\begin{aligned} (-1)^{n_\Lambda } \ b_{\lambda }\ e^{-H_\lambda (\lambda )}&= \frac{{n_\Lambda }!}{(\lambda '-\lambda )^{n_\Lambda }} \Big ({\tilde{a}}_{(\lambda ',0)} e^{-H_\lambda (\lambda ')}\nonumber \\&\qquad -\sum _{k=0}^{{n_\Lambda }-1} \sum _{j=0}^{k} (-1)^j \frac{\left( {\begin{array}{c}k\\ j\end{array}}\right) }{k!} {\tilde{a}}_{(\lambda ,j)} B_{k-j}^{G[\Delta \phi ]}(\lambda ) e^{-H_\lambda (\lambda )} (\lambda '-\lambda )^k \Big ) \nonumber \\&\qquad - \sum _{j=0}^{{n_\Lambda }-1} (-1)^j \left( {\begin{array}{c}{n_\Lambda }\\ j\end{array}}\right) {\tilde{a}}_{(\lambda ,j)} B_{{n_\Lambda }-j}^{G[\Delta \phi ]}(\lambda ) e^{-H_\lambda (\lambda )}. \end{aligned}$$

The sequence satisfies

5.5 $$\begin{aligned} \left| b_{\lambda }\right| ^2 e^{-\phi (\lambda )} \lesssim \left| b_{\lambda } e^{-H_\lambda (\lambda )}\right| ^2 \lesssim \varepsilon ^{-2{n_\Lambda }}\left( |{\tilde{a}}_{(\lambda ',0)}|^2 e^{-\phi (\lambda ')}+\sum _{j=0}^{{n_\Lambda }-1} |{\tilde{a}}_{(\lambda ,j)}|^2 e^{-\phi (\lambda )}\right) .\nonumber \\ \end{aligned}$$

We define a new sequence $a=\left\{ a_{(\lambda ,j)} \right\} _{{\lambda \in \Lambda ,\, 0\le j \le m_{\Lambda }(\lambda )-1 }} \subseteq \mathbb {C}$ by:

$$\begin{aligned}a_{(\lambda ,j)} := {\left\{ \begin{array}{ll} b_\lambda &{}\quad \text {if }\lambda \in \Lambda _{\text {max}}\text { and } j={n_\Lambda }, \\ {\tilde{a}}_{(\lambda ,j)} &{}\quad \text {otherwise.} \end{array}\right. } \end{aligned}$$

By (5.5),

5.6 $$\begin{aligned} \begin{aligned} \Vert a\Vert _{ \ell _\phi ^2(\Lambda ,\,m_{\Lambda })}^2 \lesssim \varepsilon ^{-2n_\Lambda } \Vert {\tilde{a}}\Vert _{\ell _\phi ^2({\tilde{\Lambda }},\,m_{{\tilde{\Lambda }}})}^2 <\infty . \end{aligned} \end{aligned}$$

Since $(\Lambda ,m_\Lambda )$ is an interpolating set, there exists a function $f \in \mathcal {F}_{\phi }^{2}$ such that

- $$\Vert f\Vert _{\mathcal {F}_{\phi }^{2}} \le C_{\Lambda } \Vert a\Vert _{ \ell _\phi ^2(\Lambda ,\,m_{\Lambda })}$$,^4^
, if $$\lambda \in \Lambda $$ and $$0\le j \le {\min \{{n_\Lambda }-1, m_\Lambda (\lambda )-1 \}}$$,
, if $$\lambda \in \Lambda _{\text {max}}$$.

The first two conditions, together with (5.6) yield **(Q.1)** and **(Q.2)**. To check **(Q.3)**, we let $\lambda \in \Lambda _{\text {max}}$, and note that, in terms of *f*, (5.4) reads:Applying (5.3) to the last equation we obtain

5.7 $$\begin{aligned} \begin{aligned} \partial ^{{n_\Lambda }}({fe^{-H_\lambda }})(\lambda ) = \frac{ {n_\Lambda }!}{(\lambda ' -\lambda )^{n_\Lambda }}&\Big ({\tilde{a}}_{(\lambda ',0)} e^{-H_\lambda (\lambda ')} -\sum _{k=0}^{{n_\Lambda }-1} \frac{\partial ^{k}({fe^{-H_\lambda }})(\lambda )}{k!} (\lambda '-\lambda )^k \Big ). \end{aligned}\nonumber \\ \end{aligned}$$

We apply Remark 5.1 to $F=fe^{-H_\lambda }$ and conclude from (5.2) that

$$\begin{aligned}&\left| f(\lambda ')-{\tilde{a}}_{(\lambda ',0)} \right| ^2 e^{-\phi (\lambda ')} \lesssim \left| f(\lambda ')e^{-H_\lambda (\lambda ')}-{\tilde{a}}_{(\lambda ',0)} e^{-H_\lambda (\lambda ')}\right| ^2 \\&\quad \lesssim \varepsilon ^{2({n_\Lambda }+1)} \Vert f\Vert _{L^2(B(\lambda ',1),e^{-\phi })}^2. \end{aligned}$$

Therefore,

$$\begin{aligned} \Vert f - {\tilde{a}}_{(\cdot , 0)}\Vert _{\ell _\phi ^2(\Lambda ')}^2&\lesssim \sum _{\lambda ' \in \Lambda '} \varepsilon ^{2({n_\Lambda }+1)} \Vert f\Vert _{L^2(B(\lambda ',1),e^{-\phi })}^2 \\ {}&\lesssim {\text {rel}}(\Lambda )\, \varepsilon ^{2({n_\Lambda }+1)}\,\Vert f\Vert _{\mathcal {F}_{\phi }^{2}}^2, \end{aligned}$$

which yields **(Q.3)**.

**Step 3**. We show that $\left( {\tilde{\Lambda }},m_{{\tilde{\Lambda }}}\right)$ is an interpolating set.

Let ${\tilde{a}} \in \ell _\phi ^2{({\tilde{\Lambda }},\,m_{{\tilde{\Lambda }}})}$. We define inductively ${{\tilde{a}}}^{(k)}\in \ell _\phi ^2{({\tilde{\Lambda }},\,m_{{\tilde{\Lambda }}})}$ and $f_k\in \mathcal {F}_{\phi }^{2}$. Let ${\tilde{a}}^{(1)}:={\tilde{a}}$. By Step 2, there exists $f_1\in \mathcal {F}_{\phi }^{2}$ satisfying **(Q.1)**, **(Q.2)** and **(Q.3)** for ${\tilde{a}}^{(1)}$. Let $k\ge 2$ and suppose that ${{\tilde{a}}}^{(k-1)} \in \ell _\phi ^2{({\tilde{\Lambda }},\,m_{{\tilde{\Lambda }}})}$ and $f_{k-1}\in \mathcal {F}_{\phi }^{2}$ are already defined and satisfy **(Q.1)**, **(Q.2)** and **(Q.3)** with respect to ${{\tilde{a}}}^{(k-1)}$. Define ${{\tilde{a}}}^{(k)}\in \ell _\phi ^2{({\tilde{\Lambda }},\,m_{{\tilde{\Lambda }}})}$ by

5.8 $$\begin{aligned} {{\tilde{a}}}_{(\lambda ,j)}^{(k)} := {\left\{ \begin{array}{ll} 0 &{}\quad \text {if }\lambda \in \Lambda \text { and } 0\le j \le m_{{\tilde{\Lambda }}}(\lambda )-1,\\ {{\tilde{a}}}_{(\lambda ,0)}^{(k-1)}-f_{k-1}(\lambda ) &{}\quad \text {if }\lambda \in \Lambda '\text { and } j=0.\\ \end{array}\right. } \end{aligned}$$

Since $\Vert {\tilde{a}}^{(k)}\Vert _{\ell _\phi ^2({\tilde{\Lambda }},\,m_{{\tilde{\Lambda }}})}=\Vert f_{k-1}-{{\tilde{a}}}_{(\cdot ,0)}^{(k-1)}\Vert _{\ell _\phi ^2(\Lambda ')}\le C_{\Lambda } \varepsilon ^{{n_\Lambda }+1}\Vert f_{k-1}\Vert _{\mathcal {F}_{\phi }^{2}}<\infty$, the sequence is indeed well-defined, and we can apply Step 2 for ${\tilde{a}}^{(k)}$. Let $f_k$ satisfy **(Q.1)**, **(Q.2)** and **(Q.3)** with respect to ${\tilde{a}}^{(k)}$.

The constructed sequences satisfy

$$\begin{aligned}\Vert f_{k}\Vert _{\mathcal {F}_{\phi }^{2}}\le C_\Lambda \varepsilon ^{-{n_\Lambda }}\Vert {\tilde{a}}^{(k)}\Vert _{\ell _\phi ^2({\tilde{\Lambda }},\,m_{{\tilde{\Lambda }}})}\le \varepsilon \, C_\Lambda ^{'} \Vert f_{k-1}\Vert _{\mathcal {F}_{\phi }^{2}}, \end{aligned}$$

and,

$$\begin{aligned}\Vert {\tilde{a}}^{(k)}\Vert _{\ell _\phi ^2({\tilde{\Lambda }},\,m_{{\tilde{\Lambda }}})} \le \varepsilon \, C_\Lambda ^{'}\, \Vert {\tilde{a}}^{(k-1)}\Vert _{\ell _\phi ^2{({\tilde{\Lambda }},\,m_{{\tilde{\Lambda }}})}}. \end{aligned}$$

Hence, if $\varepsilon \, C_\Lambda ^{'} < 1$, $f:=\sum _{k\in \mathbb {N}}f_k$ converges in $\mathcal {F}_{\phi }^{2}{}$.

To see that interpolates with the right values on $\Lambda ^{'}$, we observe that for each $\lambda \in \Lambda _{\text {max}}$,

$$\begin{aligned} \left| \sum _{k=1}^{N} f_k(\lambda ')-\tilde{a}^{(1)}_{(\lambda ',0)}\right| ^2 e^{-\phi (\lambda ')}&= \left| \tilde{a}^{(N +1)}_{(\lambda ',0)}\right| ^2 e^{-\phi (\lambda ')} \\&\le \Vert \tilde{a}^{(N+1)}\Vert ^2_{\ell _\phi ^2(\Lambda ')} \xrightarrow [N \rightarrow \infty ]{} 0. \end{aligned}$$

Thus, $f(\lambda ')= \tilde{a}^{(1)}_{(\lambda ',0)}$, when $\lambda \in \Lambda _{\text {max}}.$ For $\lambda \in \Lambda {\setminus }\Lambda '$ we argue as follows. Since $\sum _{k\in \mathbb {N}}f_k$ converges in $\mathcal {F}_{\phi }^{2}{}$, by Lemma 3.5, . As  for $k\ge 2$, , if $0 \le j \le m_{{\tilde{\Lambda }}}(\lambda )-1$.

We have thus shown that $({\tilde{\Lambda }},m_{{\tilde{\Lambda }}})$ is an interpolating set.

**Step 4.** We show that $D^{\pm }(\Lambda , m_{\Lambda })=D^{\pm }({\tilde{\Lambda }}, m_{{\tilde{\Lambda }}})$.

The number of points of the set $\Lambda$ in the disk of center *z* and radius *r*, counted with multiplicities, satisfies . Since $0<m \le \Delta \phi \le M$ then for any $\delta >0$, there exists $r_{\delta }$ such that if $r > r_{\delta }$, then $\sup _{z \in {\mathbb {C}}} \left| \frac{\int _{B(z, r+\varepsilon )} \Delta \phi }{\int _{B(z, r)} \Delta \phi }-1\right| <\delta$. Thus,We let $\delta \rightarrow 0$. Exchanging the roles of $\Lambda$ and ${\tilde{\Lambda }}$ we also see that $D_{\phi }^{-}\left( {\tilde{\Lambda }}, m_{{\tilde{\Lambda }}}\right) \le D_{\phi }^{-}\left( \Lambda , m_{\Lambda }\right)$. Analogously, $D_{\phi }^{+}\left( \Lambda , m_{\Lambda }\right) = D_{\phi }^{+}\left( {\tilde{\Lambda }}, m_{{\tilde{\Lambda }}}\right)$. $\square$

### Sampling

The modification of sampling sets to reduce their multiplicity is based on the following perturbation lemma.

#### Lemma 5.3

Let $\lambda ,\lambda ' \in \mathbb {C}$ and $0<\varepsilon <1/4$. If $f\in \mathcal {F}_{\phi }^{2}$ and $|\lambda '-\lambda |=\varepsilon$, there exists a constant $C_{\varepsilon }$ such that

#### Proof

We apply Theorem 3.1 on ${B(\lambda ,1)}$ and obtain $\phi (z)=h_\lambda (z)+G[\Delta \phi ](z)$. Since $h_\lambda$ is harmonic, there exists $H_\lambda :B(\lambda ,1)\longrightarrow \mathbb {C}$ holomorphic such that ${\text {Re}}{H_\lambda }=h_\lambda /2$. Therefore,Since $\partial (e^{-\overline{H_\lambda }})=\partial (\overline{e^{-H_\lambda }})=\overline{\left( \partial _{{\overline{z}}} {e^{-H_\lambda }} \right) }\equiv 0,$

5.9 $$\begin{aligned} \begin{aligned} \left| \partial ^{n_\Lambda } \left( f e^{-H_\lambda -\overline{H_\lambda }-G[\Delta \phi ]}\right) (\lambda )\right| ^2 e^{\phi (\lambda )}&= \left| \partial ^{n_\Lambda } \left( f e^{-H_\lambda -G[\Delta \phi ]}\right) (\lambda )\right| ^2 \left| e^{-\overline{H_\lambda (\lambda )}} \right| ^2 e^{\phi (\lambda )} \\&= \left| \partial ^{n_\Lambda } \left( f e^{-H_\lambda -G[\Delta \phi ]}\right) (\lambda )\right| ^2 e^{-h_\lambda (\lambda )} e^{\phi (\lambda )} \\&\lesssim \left| \partial ^{n_\Lambda } \left( f e^{-H_\lambda -G[\Delta \phi ]}\right) (\lambda )\right| ^2, \end{aligned}\end{aligned}$$

where we used that $-h_\lambda (\lambda )+\phi (\lambda )=G[\Delta \phi ](\lambda )$ is uniformly bounded, by Lemma 3.3. In addition, by Lemma 3.4,5.10We proceed to estimate $\left| (\partial ^{n_\Lambda } (f e^{-H_\lambda }))(\lambda )\right|$. The Taylor expansion for $fe^{-H_\lambda }$ of order ${n_\Lambda }$ at $\lambda$ evaluated at $\lambda '$ is

$$\begin{aligned}\left( fe^{-H_\lambda }\right) (\lambda ')=\sum _{k= 0}^{n_\Lambda } \frac{\partial ^k{(fe^{-H_\lambda })}(\lambda )}{k!}(\lambda '-\lambda )^k+E_{n_\Lambda }(\lambda '). \end{aligned}$$

Hence,

$$\begin{aligned}&{\partial ^{n_\Lambda } \left( fe^{-H_\lambda }\right) }(\lambda ) \\&\quad =\frac{{n_\Lambda }!}{(\lambda '-\lambda )^{n_\Lambda }}{(fe^{-H_\lambda })}(\lambda ') - \frac{{n_\Lambda }!}{(\lambda '-\lambda )^{n_\Lambda }} \sum _{k=0}^{{n_\Lambda }-1} \frac{{\partial ^k (fe^{-H_\lambda })}(\lambda )}{k!}(\lambda '-\lambda )^k\\&\qquad -\frac{{n_\Lambda }!}{(\lambda '-\lambda )^{n_\Lambda }}E_{n_\Lambda }(\lambda '), \end{aligned}$$

and for $$|\lambda '-\lambda |=\varepsilon $$,

$$\begin{aligned} \left| {\partial ^{n_\Lambda }(fe^{-H_\lambda })}(\lambda )\right| ^2&\lesssim C_{\varepsilon , {n_\Lambda }} \left( |{(fe^{-H_\lambda })}(\lambda ')|^2 + \sum _{k=0}^{{n_\Lambda }-1} |\partial ^k(fe^{-H_\lambda })(\lambda )|^2\right) \\&\qquad +\frac{|E_{n_\Lambda }(\lambda ')|^2}{\varepsilon ^{2n}}. \end{aligned}$$

We use the last estimate, together with Lemma 3.4 and the fact that $\phi (z)-h_\lambda (z)$ is bounded, and (5.10) to obtain:Moreover, if $|\lambda '-\lambda |=\varepsilon$, the remainder of the Taylor expansion satisfies (5.1) with $F=f e^{-H_\lambda }$, and, therefore,The claim follows since $\varepsilon ^2 < \varepsilon$. $\square$

We can now modify a sampling set to reduce its maximal multiplicity without altering its sampling property.

#### Proposition 5.4

Let $\left( \Lambda , m_{\Lambda }\right)$ be a separated set with multiplicities that is compatible with $\phi$. Assume that $(\Lambda ,m_{\Lambda })$ is sampling for $\mathcal {F}_{\phi }^{2}(\mathbb {C})$, and that $\sup _{\lambda \in \Lambda } m_{\Lambda }(\lambda ) = {n_\Lambda }+1 \ge 2$. Then there exists a separated set with multiplicities $({\tilde{\Lambda }}, m_{{\tilde{\Lambda }}})$ that is sampling for $\mathcal {F}_{\phi }^{2}$, and such that $\sup _{\lambda \in {\tilde{\Lambda }}} m_{{\tilde{\Lambda }}}(\lambda )={n_\Lambda }$ and $D^{\pm }(\Lambda , m_{\Lambda })=D^{\pm }({\tilde{\Lambda }}, m_{{\tilde{\Lambda }}})$.

#### Proof

Since $(\Lambda ,m_{\Lambda })$ is a sampling set for $\mathcal {F}_{\phi }^{2}(\mathbb {C})$,5.11Let $0<\varepsilon <\min \{\rho (\Lambda )/2,\ 1/4\}$ and consider $\Lambda _{\text {max}}$, $\Lambda ^{\prime }$, ${\tilde{\Lambda }}$ and $m_{{\tilde{\Lambda }}}$ as in the proof of Proposition 5.2. Hence, $D^{\pm }(\Lambda , m_\Lambda )=D^{\pm }({\tilde{\Lambda }}, m_{{\tilde{\Lambda }}})$, and ${\tilde{\Lambda }}$ is separated. As a consequence, $({\tilde{\Lambda }}, m_{{\tilde{\Lambda }}})$ satisfies an upper sampling bound. We now show that if $\varepsilon$ is small enough, a lower sampling bound also holds for $({\tilde{\Lambda }}, m_{{\tilde{\Lambda }}})$.

For $\lambda \in \Lambda _{\text {max}}$, we have $m_{{\tilde{\Lambda }}}(\lambda )=n_\Lambda =m_{\Lambda }(\lambda )-1$ and, by Lemma 5.3,On the other hand, for $\lambda \in \Lambda {\setminus }\Lambda _{\text {max}}$, $m_{\Lambda }(\lambda )=m_{{\tilde{\Lambda }}}(\lambda )$, and the same equation is trivially true. Therefore, by (5.11),5.12Thus, taking $\varepsilon$ small enough, the term $\varepsilon {\text {rel}}(\Lambda ) \Vert f\Vert ^2_{\mathcal {F}_{\phi }^{2}}$ can be absorbed into the left-hand side, yielding the desired lower sampling bound. $\square$

## Proof of the Main Results

### Proof of Theorem A

Suppose that $(\Lambda , m_\Lambda )$ is set of interpolation for $\mathcal {F}_{\phi }^{2}$. Then the unweighted set $\Lambda$ is also a set of interpolation for $\mathcal {F}_{\phi }^{2}$, and therefore separated; see, e.g., [19, Prop. 3]. Repeated application of Proposition 5.2 shows that there exists a set of interpolation ${\tilde{\Lambda }}\subseteq \mathbb {C}$ without multiplicity such that $D_\phi ^{+}(\Lambda , m_\Lambda )=D_\phi ^{+}({\tilde{\Lambda }})$. As shown in [19, Theorem 2], ${\tilde{\Lambda }}$ must satisfy $D_\phi ^{+}({\tilde{\Lambda }}) < 1/\pi {}$. Note that in [19], the space $\mathcal {F}_{\phi }^{2}$ is denoted $\mathcal {F}_{2\phi }^{2}$.

Towards the sufficiency of the density conditions, let $(\Lambda , m_\Lambda )$ be a separated set with multiplicities, satisfying $D_{\phi }^{+}(\Lambda ,m_{\Lambda }) < 1/\pi {}$. Hence, for some $\varepsilon > 0$ we have $D_{\phi }^{+}(\Lambda ,m_{\Lambda }) < 1/\pi {} - \varepsilon$. Thus, for a certain $R > 0$ and for all $z\in \mathbb {C}$,where the bar in the integral denotes an average (division by the total measure). Let $\chi _{R}=\left( \pi R^{2}\right) ^{-1} 1_{B(0, R)}$, ${\tilde{\phi }}=\phi \star \chi _R$, and $\nu _{\Lambda } :=\sum _{\lambda \in \Lambda } m_\Lambda (\lambda ) \cdot \delta _{\lambda }$. ThenWe wish to apply Proposition 4.2, with the roles of the weights $\phi$ and ${\tilde{\phi }}$ interchanged. The density condition (4.9) is verified for ${\tilde{\phi }}$, because of the previous equation. In addition, $\Delta {\tilde{\phi }}$ is continuous, and $\Delta {\tilde{\phi }}$ is bounded above and below with the same constants that bound $\Delta \phi$. Moreover, we claim that

6.1 $$\begin{aligned} \Vert \partial ^{j} (\phi - {\tilde{\phi }})\Vert _\infty < \infty , \qquad 0 \le j \le n_\Lambda . \end{aligned}$$

To see this, let $z_0 \in {\mathbb {C}}$ and let

6.2 $$\begin{aligned} \phi (w) = h(w) + G[\Delta \phi ](w), \qquad w \in B(z_0,R+1), \end{aligned}$$

be the Riesz decomposition of $\phi$ on $B(z_0,R+1)$, as given by Theorem 3.1. Let $z \in B(z_0,1)$. We average (6.2) over *B*(*z*, *R*) and use the mean value property for harmonic functions to conclude

$$\begin{aligned} {\tilde{\phi }}(z) = h(z) + G[\Delta \phi ]*\chi _{R}(z), \end{aligned}$$

and, therefore,

$$\begin{aligned} \phi (z)-{\tilde{\phi }}(z) = G[\Delta \phi ](z) - G[\Delta \phi ]*\chi _{R}(z), \qquad z \in B_1(z_0). \end{aligned}$$

The desired number of derivatives of each term on the right-hand side of the previous equation is bounded independently of $z_0$ by Lemma 3.3, (3.4) (the bound does depend on *R*). Hence, (6.1) follows. We can therefore apply Proposition 4.2, with the roles of the weights $\phi$ and ${\tilde{\phi }}$ interchanged, and conclude that $\left( \Lambda , m_\Lambda \right)$ is an interpolating set for $\mathcal {F}_{\phi }^{2}$ (indeed, $\mathcal {F}_{\phi }^{2}=\mathcal {F}_{{\tilde{\phi }}}^{2}$, and the interpolation property is considered with respect to ${\bar{\partial }}^*_\phi$.) $\square$

### Proof of Theorem B

Suppose that $(\Lambda , m_{\Lambda })$ is sampling for $\mathcal {F}_{\phi }^{2}$. Then, for some constant $B>0$,The upper sampling bound without multiplicities already implies that $\Lambda$ is relatively separated; see, e.g., [19, Prop. 1]. The existence of a separated set $\Lambda '\subseteq \Lambda$ which is also sampling for $\mathcal {F}_{\phi }^{2}$ follows from standard arguments (see for instance [3, Theorem 3.7]). Without loss of generality we assume that $\Lambda$ is already separated. By repeated application of Proposition 5.4, there is a sampling set ${\tilde{\Lambda }}\subseteq \mathbb {C}$ without multiplicities, such that $D_\phi ^{-}(\Lambda , m_\Lambda )=D_\phi ^{-}({\tilde{\Lambda }})$. As shown in [19, Theorem 1], ${\tilde{\Lambda }}$ must satisfy $D_\phi ^{-}({\tilde{\Lambda }}) > 1/\pi {}$.

Conversely, suppose that $(\Lambda , m_\Lambda )$ is a relatively separated set with multiplicities containing a separated subset $\Lambda ^\prime$, satisfying . The upper sampling bound follows from the fact that $\Lambda$ is relatively separated and Lemma 3.5. For the lower sampling bound we assume without loss of generality that $\Lambda =\Lambda ^\prime$. Proceeding as in the proof of Theorem A, we consider ${\tilde{\phi }} = \phi \star \chi _R$, for some large $R>0$ and conclude from Proposition 4.3, that $\left( \Lambda , m_\Lambda \right)$ is a sampling set for $\mathcal {F}_{{\tilde{\phi }}}^{2}$. By (6.1), $\mathcal {F}_{\phi }^{2}$ and $\mathcal {F}_{{\tilde{\phi }}}^{2}$ contain the same functions and have equivalent norms. Moreover, for each $f\in \mathcal {F}_{\phi }^{2}$, we combine (4.8) and (6.1) to conclude thatThis gives the desired bound. $\square$

## Appendix A. Bell Polynomials and Faà di Bruno’s Formula

The Bell polynomials appear naturally when calculating the *n*-th derivative of a composite function. For a deeper discussion of this topic we refer the reader to [15, pp. 95–98] and for the proofs [4].

### A.1. Bell Polynomials

Let $n, k \in {\mathbb {N}}$ such that $k \le n$. The partial exponential Bell polynomials are a collection of polynomials given by

$$\begin{aligned} B_{n, k}\left( x_{1}, x_{2}, \ldots , x_{n-k+1}\right)&=\sum \frac{n !}{m_{1} ! m_{2} ! \cdots m_{n-k+1} !}\left( \frac{x_{1}}{1 !}\right) ^{m_{1}}\left( \frac{x_{2}}{2 !}\right) ^{m_{2}}\\&\quad \cdots \left( \frac{x_{n-k+1}}{(n-k+1) !}\right) ^{m_{n-k+1}} \end{aligned}$$

where the sum is taken over all sequences $m_{1}, m_{2}, m_{3}, \ldots , m_{n-k+1}$ of non-negative integers such that the following conditions are satisfied:

$$\begin{aligned}\begin{array}{l}{m_{1}+m_{2}+\cdots +m_{n-k+1}=k} \\ {m_{1}+2 m_{2}+3 m_{3}+\cdots +(n-k+1) m_{n-k+1}=n}\end{array} . \end{aligned}$$

If $n \ge 1$, the sum

A.1 $$\begin{aligned} B_{n}\left( x_{1}, \ldots , x_{n}\right) =\sum _{k=1}^{n} B_{n, k}\left( x_{1}, x_{2}, \ldots , x_{n-k+1}\right) \end{aligned}$$

is called the $n-$th complete exponential Bell polynomial. If $n=0$, then

$$\begin{aligned} B_{0} := 1. \end{aligned}$$

#### Remark A.1

The partial Bell polynomials $B_{n,k}$ are homogeneous polynomials of degree k, therefore it follows from (A.1) that the $n-$th complete Bell polynomial $B_n$ has no constant term as long as $n \ge 1$.

The complete Bell polynomials satisfy the binomial type relation:

A.2 $$\begin{aligned} B_{n}\left( x_{1}+y_{1}, \ldots , x_{n}+y_{n}\right) =\sum _{i=0}^{n}\left( {\begin{array}{c}n\\ i\end{array}}\right) B_{n-i}\left( x_{1}, \ldots , x_{n-i}\right) B_{i}\left( y_{1}, \ldots , y_{i}\right) .\quad \quad \quad \end{aligned}$$

### A.2. Chain Rule for Higher Derivatives

Suppose that *f* and *r* are *n*-times differentiable functions, then

$$\begin{aligned}\frac{d^{n}}{d x^{n}} f(r(x))=\sum _{k=1}^{n} f^{(k)}(r(x)) \cdot B_{n, k}\left( r^{\prime }(x), r^{\prime \prime }(x), \ldots , r^{(n-k+1)}(x)\right) . \end{aligned}$$

In what follows, we use the abbreviation

A.3 $$\begin{aligned} B_{n, k}^{r}(x)&:= B_{n, k}\left( r^{\prime }(x), r^{\prime \prime }(x), \ldots , r^{(n-k+1)}(x)\right) , \end{aligned}$$

A.4 $$\begin{aligned} B_{n}^{r}(x)&:= B_{n}\left( r^{\prime }(x), r^{\prime \prime }(x), \ldots , r^{(n)}(x)\right) . \end{aligned}$$

#### Remark A.2

Suppose that *f* and *r* are functions both differentiable *n* times. With the notation introduced in (A.3) and (A.4), (A.2) can be written as

A.5 $$\begin{aligned} B_{n}^{f+g}=\sum _{i=0}^{n}\left( {\begin{array}{c}n\\ i\end{array}}\right) B_{n-i}^{f} B_{i}^g. \end{aligned}$$

One special case of $\frac{d^{n}}{d x^{n}} f(r(x))$ is when $f(t)=e^t$, obtaining:

A.6 $$\begin{aligned} \frac{d^{n}}{d x^{n}} e^{r(x)}&=\sum _{k=1}^{n} e^{r(x)} \cdot B_{n, k}^{r}(x) = e^{r(x)} \sum _{k=1}^{n} B_{n, k}^{r}(x) = e^{r(x)} B_{n}^{r}(x). \end{aligned}$$

### A.3. Proof of Equation 4.7

We want to prove that the coefficients $k_j$ defined by (4.7) for $j\in \{0,\dots ,m_\Lambda (\lambda )-1\}$ solve the recursive equation (4.6). We proceed by induction on *j*. The case $j=0$ is clear. Suppose that for every $0\le l< j$,

$$\begin{aligned} k_l = (-1)^l c_{l} - {\sum _{m=0}^{l-1} \left( {\begin{array}{c}l\\ m\end{array}}\right) \ k_{m} }\ B_{l-m}^{G_{\lambda }-\phi }(\lambda ), \end{aligned}$$

or, equivalently,

$$\begin{aligned} (-1)^l c_{l} = {\sum _{m=0}^{l} \left( {\begin{array}{c}l\\ m\end{array}}\right) \ k_{m} }\ B_{l-m}^{G_{\lambda }-\phi }(\lambda ). \end{aligned}$$

We use the inductive hypothesis, together with fact that $B_{0}^{\phi -G_{\lambda }} \equiv 1 \equiv B_{0}^{G_\lambda -\phi }$, and (A.5) to compute:

$$\begin{aligned} k_j ={}&(-1)^j c_{j} + {\sum _{l=0}^{j-1} (-1)^l \left( {\begin{array}{c}j\\ l\end{array}}\right) \ c_{l} }\ B_{j-l}^{\phi -G_\lambda }(\lambda ) \\ ={}&(-1)^j c_{j} + {\sum _{l=0}^{j-1} \left( {\begin{array}{c}j\\ l\end{array}}\right) \ \left( {\sum _{m=0}^{l} \left( {\begin{array}{c}l\\ m\end{array}}\right) \ k_{m} }\ B_{l-m}^{G_\lambda -\phi }(\lambda ) \right) }\ B_{j-l}^{\phi - G_\lambda }(\lambda ) \\ ={}&(-1)^j c_{j} + {\sum _{l=0}^{j} \left( {\begin{array}{c}j\\ l\end{array}}\right) \ \left( {\sum _{m=0}^{l} \left( {\begin{array}{c}l\\ m\end{array}}\right) \ k_{m} }\ B_{l-m}^{G_\lambda -\phi }(\lambda ) \right) }\ B_{j-l}^{\phi -G_\lambda }(\lambda )\ \\&-{\sum _{m=0}^{j} \left( {\begin{array}{c}j\\ m\end{array}}\right) \ k_{m} }\ B_{j-m}^{G_\lambda -\phi }(\lambda ) \\ ={}&(-1)^j c_{j} + {\sum _{m=0}^{j} \left( {\begin{array}{c}j\\ m\end{array}}\right) \ k_{m}\ \left( {\sum _{l=m}^j \left( {\begin{array}{c}j-m\\ j-l\end{array}}\right) \ B_{(j-m)-(j-l)}^{G_\lambda -\phi }(\lambda )\ B_{j-l}^{\phi - G_\lambda }(\lambda ) }\right) \ } \\&-{\sum _{m=0}^{j} \left( {\begin{array}{c}j\\ m\end{array}}\right) \ k_{m} }\ B_{j-m}^{G_\lambda -\phi }(\lambda ) \\ ={}&(-1)^j c_{j} + {\sum _{m=0}^{j} \left( {\begin{array}{c}j\\ m\end{array}}\right) \ k_{m}\ \left( {\sum _{l=0}^{j-m} \left( {\begin{array}{c}j-m\\ l\end{array}}\right) \ B_{(j-m)-l}^{G_\lambda -\phi }(\lambda )\ B_{l}^{\phi - G_\lambda }(\lambda ) }\right) \ } \\&-{\sum _{m=0}^{j} \left( {\begin{array}{c}j\\ m\end{array}}\right) \ k_{m} }\ B_{j-m}^{G_\lambda -\phi }(\lambda ) \\ ={}&(-1)^j c_{j} + k_j + {\sum _{m=0}^{j-1} \left( {\begin{array}{c}j\\ m\end{array}}\right) \ k_{m}\ B_{j-m}^{0} (\lambda )} \ - {\sum _{m=0}^{j-1} \left( {\begin{array}{c}j\\ m\end{array}}\right) \ k_{m} }\ B_{j-m}^{G_\lambda -\phi }(\lambda ) \ -\ k_j. \end{aligned}$$

Finally, by Remark (A.1), $B_{j-m}^{0}=0$ if $j-m >0$ and we obtain:

$$\begin{aligned} k_j = (-1)^j c_j - {\sum _{m=0}^{j-1} \left( {\begin{array}{c}j\\ m\end{array}}\right) \ k_{m} }\ B_{j-m}^{G_\lambda -\phi }(\lambda ), \end{aligned}$$

as desired.

## Footnotes

1 See also [17] for necessary density conditions for sampling and interpolation in Paley–Wiener spaces with arbitrary spectra.

2 In principle, $\Delta \psi$ is a distribution; Hörmander’s $L^2$-estimate must be combined with a regularization argument to yield the conclusion.

3 To unload the notation, we write $\Vert f\Vert _{\ell ^2_\phi (\Lambda )}$ instead of $\Vert f|\Lambda \Vert _{\ell ^2_\phi (\Lambda )}$.

4 As in the unweighted case, and with the same argument, the interpolation problem, if solvable, can be solved with norm control.

## References

[1] Adcock B, Gataric M, Hansen AC (2017). Density theorems for nonuniform sampling of bandlimited functions using derivatives or bunched measurements. J. Fourier Anal. Appl..

[2] Antony Selvan A, Radha R (2016). Separation of zeros, a Hermite interpolation based and a frame based reconstruction algorithm for bandlimited functions. Sampl. Theory Sig. Image Process..

[3] Ascensi G, Bruna J (2009). Model space results for the Gabor and wavelet transforms. IEEE Trans. Inform. Theory.

[4] Bell ET (1934). Exponential polynomials. Ann. Math..

[5] Berndtsson B, Ortega Cerdà J (1995). On interpolation and sampling in Hilbert spaces of analytic functions. J. Reine Angew. Math..

[6] Beurling, A.: Local harmonic analysis with some applications to differential operators. In: Some Recent Advances in the Basic Sciences, Vol. 1 (Proc. Annual Sci. Conf., Belfer Grad. School Sci., Yeshiva Univ., New York, 1962–1964), pp. 109–125. Belfer Graduate School of Science, Yeshiva Univ., New York (1966)

[7] Beurling, A.: The collected works of Arne Beurling. Vol. 1. In: Carleson, L., Malliavin, P., Neuberger, J., Wermer, J. (eds.) Complex Analysis. Contemporary Mathematicians, Birkhäuser Boston Inc, Boston (1989)

[8] Borichev A, Hartmann A, Kellay K, Massaneda X (2017). Geometric conditions for multiple sampling and interpolation in the Fock space. Adv. Math..

[9] Brekke S, Seip K (1993). Density theorems for sampling and interpolation in the Bargmann–Fock space. III. Math. Scand..

[10] Daubechies I, Grossmann A (1988). Frames in the Bargmann Hilbert space of entire functions. Comm. Pure Appl. Math..

[11] Gilbarg, D., Trudinger, N.S.: Elliptic Partial Differential Equations of Second Order. Classics in Mathematics. Springer, Berlin, 2001. Reprint of the 1998 edition (1998)

[12] Gröchenig K, Romero JL, Stöckler J (2020). Sharp results on sampling with derivatives in shift-invariant spaces and multi-window Gabor frames. Constr. Approx..

[13] Grohs P, Rathmair M (2019). Stable Gabor phase retrieval and spectral clustering. Comm. Pure Appl. Math..

[14] Hayman, W.K., Kennedy, P.B.: Subharmonic functions. Vol. I. Academic Press, Harcourt Brace Jovanovich, Publishers, London, New York, 1976. London Mathematical Society Monographs, No. 9 (1976)

[15] Hazewinkel, M. (ed.): Encyclopaedia of Mathematics. Supplement. Vol. I. Kluwer Academic Publishers, Dordrecht (1997)

[16] Hörmander, L.: An Introduction to Complex Analysis in Several Variables. D. Van Nostrand Co., Inc., Princeton, Toronto, London (1966)

[17] Landau HJ (1967). Necessary density conditions for sampling and interpolation of certain entire functions. Acta Math..

[18] Lyubarskiĭ, Y.I.: Frames in the Bargmann space of entire functions. In: Entire and Subharmonic Functions, pp. 167–180. Adv. Soviet Math. 11. Amer. Math. Soc., Providence (1992)

[19] Ortega-Cerdà J, Seip K (1998). Beurling-type density theorems for weighted $$L^p$$ spaces of entire functions. J. Anal. Math..

[20] Ransford, T.: Potential Theory in the Complex Plane. London Mathematical Society Student Texts, vol. 28. Cambridge University Press, Cambridge (1995)

[21] Razafinjatovo HN (1995). Iterative reconstructions in irregular sampling with derivatives. J. Fourier Anal. Appl..

[22] Saff, E.B., Totik, V.: Logarithmic potentials with external fields, volume 316 of Grundlehren der Mathematischen Wissenschaften [Fundamental Principles of Mathematical Sciences]. Springer, Berlin, 1997. Appendix B by Thomas Bloom (1997)

[23] Seip K (1992). Density theorems for sampling and interpolation in the Bargmann–Fock space. Bull. Am. Math. Soc. (N.S.).

[24] Seip K, Wallstén R (1992). Density theorems for sampling and interpolation in the Bargmann–Fock space. II. J. Reine Angew. Math..

[25] Yu, X.: Lecture Notes in Intermediate PDE. https://web.archive.org/web/20200220142540/https://www.math.ualberta.ca/~xinweiyu/527.1.11f/lec11.pdf

[26] Zhu, K.: Analysis on Fock Spaces. Graduate Texts in Mathematics, vol. 263. Springer, New York (2012)

